# Facial palsy after embolization with Squid® 12

**DOI:** 10.1186/s12883-021-02064-4

**Published:** 2021-01-30

**Authors:** Santiago Moreno-Paredes, Laura Rodríguez-Alcalá, Juan Martín-Lagos Martínez, Nicolás Müller Locatelli, Cristina Vázquez López, José Luis Vargas Fernández, Álvaro Cabrera Peña

**Affiliations:** 1 Department of Otorhinolaryngology, Hospital Clínico Universitario San Cecilio, Granada, Spain; 2 Department of Interventional Radiology, Hospital Clínico Universitario San Cecilio, Granada, Spain

**Keywords:** Facial Palsy, Squid 12, Ethylene-vinyl alcohol copolymer, Embolization, Neck paraganglioma

## Abstract

Endovascular procedures with liquid embolic agents such as ethylene-vinyl alcohol (EVOH) copolymers are indicated before surgical treatment of cervical paraganglioma. Consequently, these agents are now available as low viscosity formulations, one of which is Squid 12, which are demonstrating superior vascular penetration. Cases of facial paralysis secondary to embolization of cervical vascular lesions with classic embolic agents have been reported in the English literature, however, this complication has not been described with new generation options such as Squid 12.

We describe the case of a 43-year-old patient with a left neck carotid paraganglioma. Embolization was performed under general anaesthesia before surgical excision. In the immediate postoperative period, the patient developed total left facial palsy. Since the imaging tests (Computed Tomography (CT) and Magnetic Resonance Imaging (MRI)) and neurological examination showed no involvement of additional cranial nerves (CN), we hypothesise that the main cause of this complication is ischemia of the vasa nervorum of CN VII secondary to embolization. Almost six months later, the patient continues to present total facial paralysis (Grade VI House-Brackmann facial paralysis scale), and palsy of the left CN X and XII as a complication secondary to surgical resection of the paraganglioma.

This case is relevant since it is the first clinical case of permanent facial paralysis secondary to embolization with Squid 12.

## Background

The choice of embolic agent for embolization of vascular head and neck tumours is challenging since the vascular patterns of tumours that are optimal candidates for this agent have not yet been defined [1]. However, preoperative endovascular embolization has become an important step in the treatment of head and neck hypervascular tumours. This procedure (described for the first time by Borges et al [2]) has been shown to reduce operative duration, intraoperative blood loss and, therefore, morbidity and mortality.

Squid® (Balt Extrusion, Montmorency, France) is a copolymer of EVOH dissolved in a DMSO (dimethyl sulfoxide) solution with suspended micronized Tantalum powder for radio-opacity. While Squid 18 has similar properties to Onyx®, Squid 12 is innovative in terms of viscosity. A less viscous product, Squid 12 is more fluid than other agents. Studies show that this property enhances penetration of the tumoral parenchyma and diffusion of the embolic agent in parts of the tumour further from the puncture site [3–5]. Although embolic agents have improved, they can still cause serious postoperative complications. Specifically, the advantage of Onyx® for deep penetration of intratumoral vessels must be weighed against the risk of cranial neuropathy [3]. This diffusion through the tumour vasculature is superior with the new Squid 12. The studies in the English consider it a safe embolic agent with satisfactory obliteration rate [6]. The complications described so far have been minor, consisting of one case of transient facial paralysis with a duration less than 2 weeks with corticosteroids [7]; and another case of hypoglossal nerve palsy (most likely due to an occlusion of a small vasa nervorum), which was partially resolved by administration of steroids [8].

## Case presentation

**Fig. 1:**
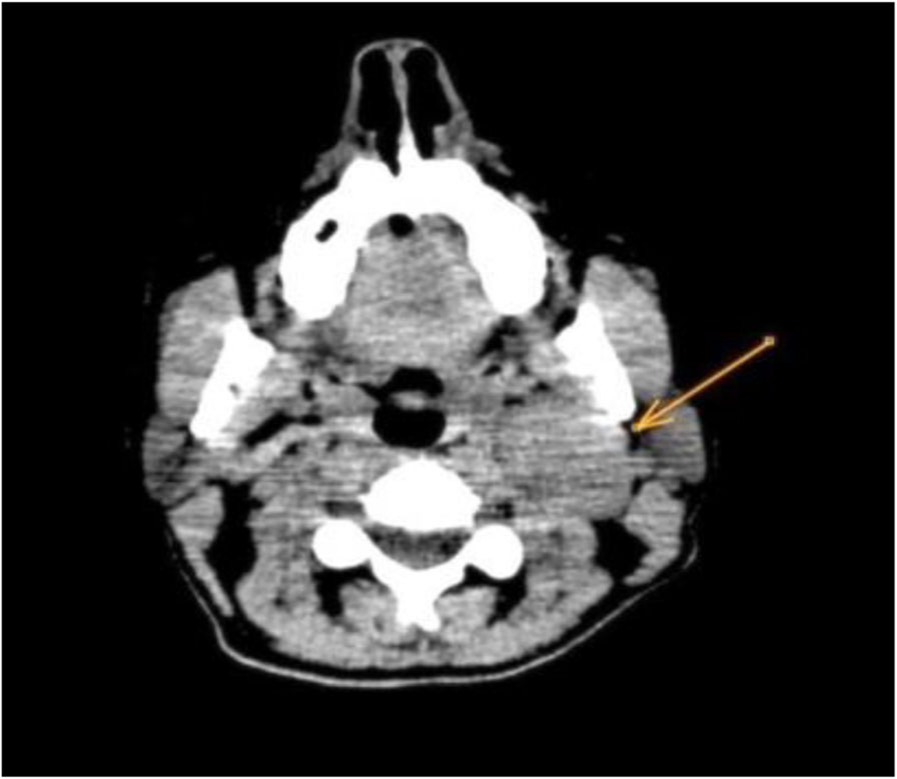
CT scan showing a left neck mass (arrow) compatible with carotid glomus

**Fig. 2:**
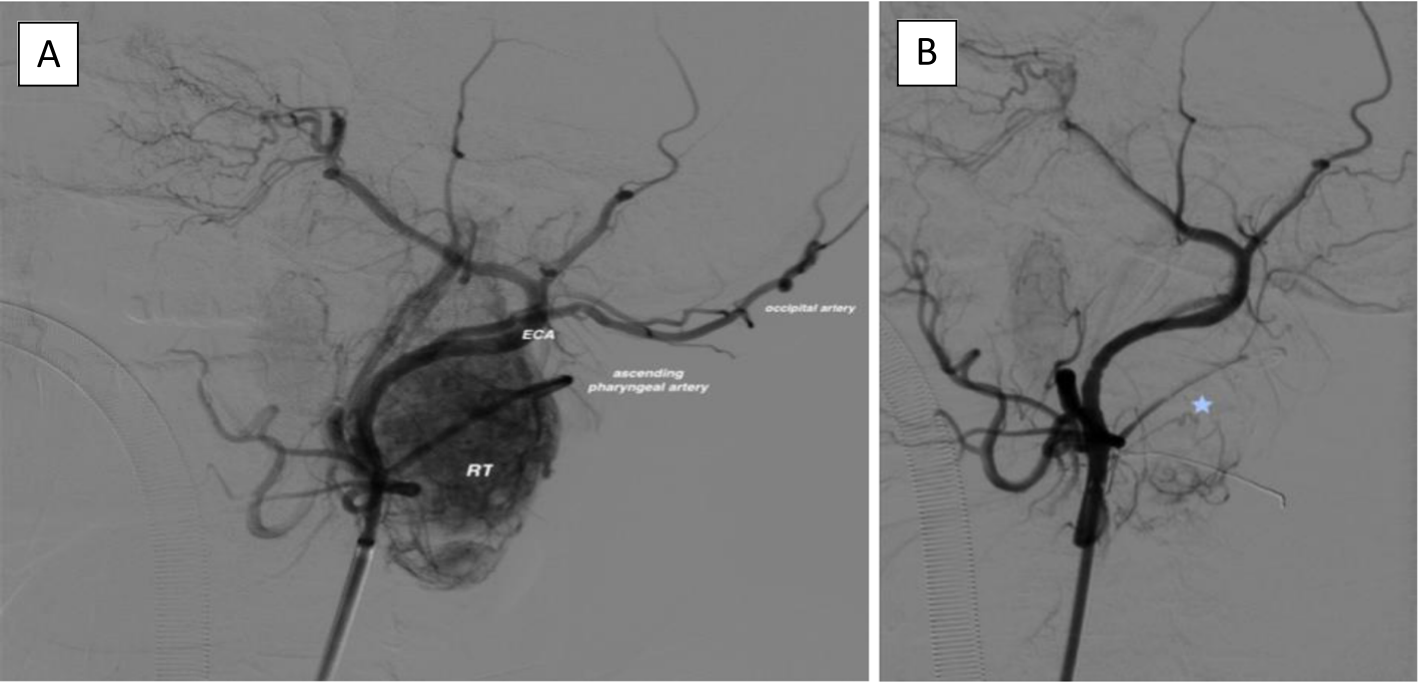
**a** Pre-embolization angiography. The ECA (external carotid artery) and its branches are visualized, with the ascending pharyngeal as the main feeding vessel. RT: tumor tissue enhancement. **b** Post-embolization angiography with decreased tumor hypervascularization (asterisk)

We describe the case of a 43-year-old woman diagnosed with cervical paraganglioma as a casual finding in computed tomography. Her medical history included sinusopathy complicated by sinus thrombosis and bilateral optic neuropathy with no clinical signs of effect on the facial or other cranial nerves. A laryngeal examination by the otolaryngologist two months earlier was normal, revealing no abnormal findings. The CT image shows a 32x26x41mm carotid paraganglioma (Fig. 1) between the internal jugular vein and the external carotid artery (Shamblin classification I/II). The patient consented to surgery with preoperative embolization. Under anesthetic sedation, the procedure was performed with a puncture in the left femoral artery, the Radifocus® Introducer II (5Fr) introducer is placed. The Optitorque™ Simmons Sidewinder I (4Fr) angiographic catheter and the hydrophilic guide Terumo® M Wire was avanzed to external carotid. With the injection of contrast medium, the tissue reinforcement of the glomus tumor is identified, showing the main pedicles dependent on the occipital, ascending pharyngeal, maxillary and superior thyroid arteries, and neoforming vessels of the ascending pharyngeal artery. With the coaxial technique, the Progreat® guide microcatheter (2.7Fr/2.8Fr) is advanced to the ascending pharyngeal artery, corroborating the vascular dependence with the injection of contrast medium and the selective embolization of the tumor, with good final angiographic results (Fig. 2).

During the immediate postoperative period, the patient developed sudden onset left facial paralysis. Our examination revealed Grade III left facial paralysis (House-Brackmann Grading System) with normal sensitivity. No other loss of neurological function was detected.

**Fig. 3:**
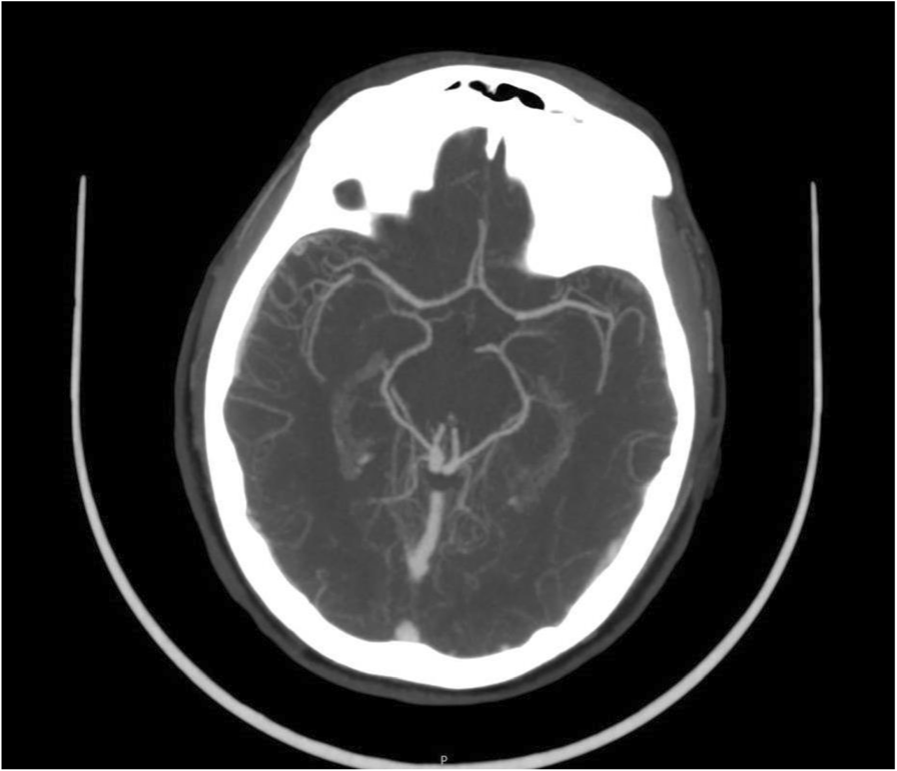
MRI angiography. Normal Willis polygon

The stroke code was activated in response to the clinical examination and vascular procedure performed. Computed tomography(CT)perfusion, and an MR angiography of the supra-aortic vessels (Figs. 2 and 3) showed no acute ischemic lesions in the supra-aortic vessels or Willis polygon. The patient was treated with high doses of corticosteroids (methylprednisolone 1.5 mg/kg/day); however, it progressed to total facial paralysis in less than 24 hours.

The surgeon completed the resection of the paraganglioma three days after the embolization, but could not avoid damaging the vagus nerve (CN X), resulting in recurrent left laryngeal nerve paralysis. The surgery also affected CN IX, XI, and XII, eliminated the gag reflex, caused hypoesthesia of the left side of the face and pharynx, velopalatine asymmetry and motor dysfunction of the tongue. Five days after the operation, the patient still presented Grade V left-side facial paralysis and the left side of the vocal chords were paralysed with signs of acute laryngeal compensation. However, the motor tone of the tongue had recovered completely. Episodes of aspiration were observed in the volume-viscosity swallow test (V-VST). Rehabilitation and inserting a nasogastric tube as an initial measure, although finally, because of the deteriorating situation, it was decided that our Dysphagia Unit should perform a percutaneous gastrostomy.

The patient continues to suffer from facial paralysis, but to a lesser degree on the House-Brackmann scale (IV): lack of lip seal, facial asymmetry at rest and poor facial movements, without synkinesis.

**Fig. 4:**
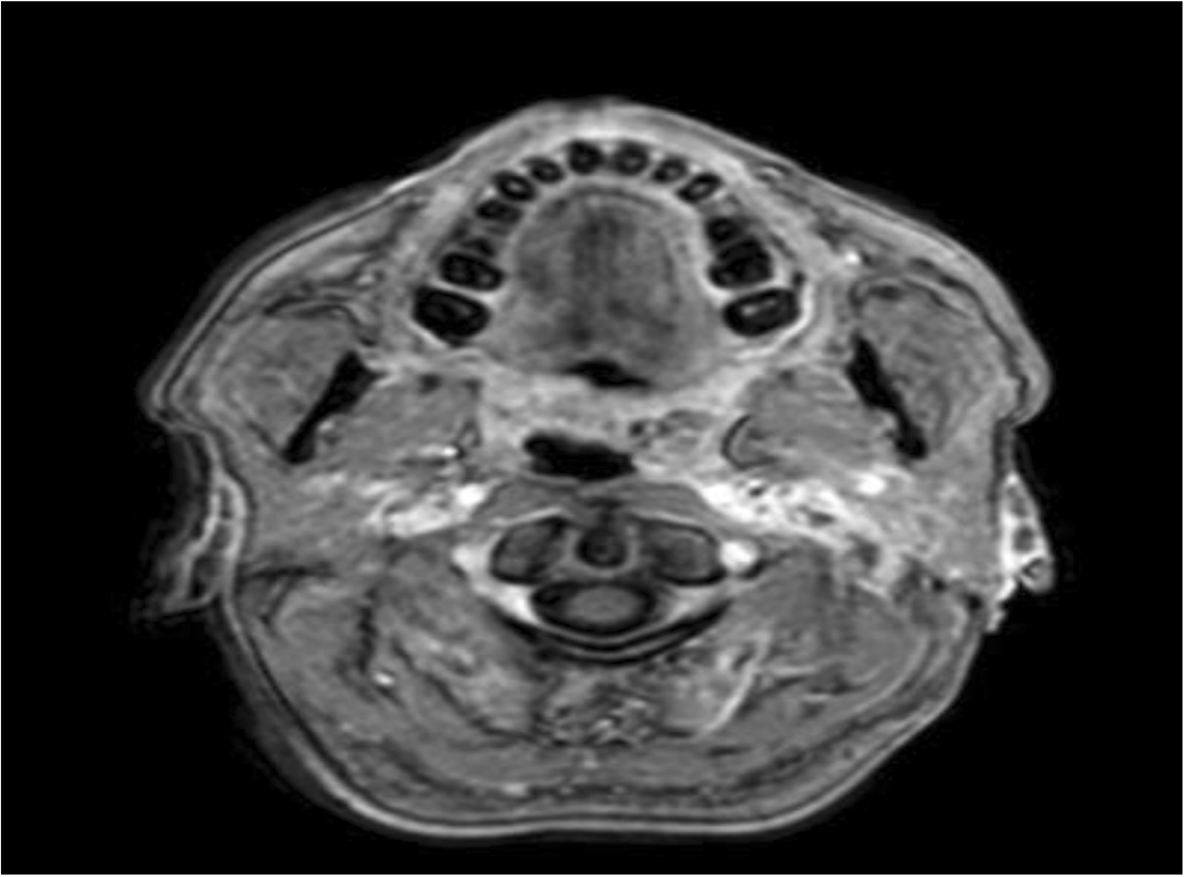
T1wMRI with post-surgical cervical changes, vascular and nerve compression

MRI performed in the immediate postoperative period (Fig. 4), showed no lesions of the facial nerve and, therefore, reconstructive surgery of the facial nerve was rejected.

## Discussion and conclusions

Facial palsy is a rare complication in patients undergoing arterial embolization. There is a theoretical estimated 6% risk of harbouring an anatomical vascular pattern that may expose the facial nerve to greater risk during embolisation [1, 3–5]. Furthermore, deep penetration of intratumoral vessels must be weighed against the risk of cranial neuropathy.

Sudden onset facial palsy subsequent to an endovascular procedure should be treated as a major complication. All patients should be apprised of this risk before undergoing this type of procedure because of its psychological and social impact. Squid 12 is considered a safe liquid embolic agent for this type of procedure.

We have described an initial case of permanent facial paralysis after the use of Squid 12 when embolising vascular tumours of the head and neck. We believe that due to the complex vascular anatomy of the area, it is likely that small vasa nervorum were occluded when diffusing the embolizing agent, since there were no difficulties during or after the technique. This vasa nervorum occlusion could have happened on the one hand, in the intrapetrosal course from the Fallopian canal, where the facial nerve is supplied by the petrous branch of the middle meningeal artery and by the stylomastoid branch of the occipital artery, which sometimes originates directly from the external carotid; and on the other hand, in the extrapetrosal portion of the facial nerve, that first receives irrigation from branches of the occipital and posterior auricular arteries at the base of the skull, and subsequently at the level of the parotid gland and outside of it, where vessels come from the superficial temporal for the temporofacial trunk and from the facial for the cervicofacial trunk [9].

If we compare the Squid 12 with the Onyx, are very similar in terms of composition. However, that lower viscosity described in the Squid 12 literature, can favor penetration in all vascular pedicles and their more distal branches, affecting irrigation vascular of the facial nerve in this case. The persistence of the nerve injury could also be favored by postoperative changes on MRI (Fig. 4).

This case shows that cranial nerve palsy is a possible complication of the procedure even with the new embolic agents such as Squid 12, therefore an accurate risk-benefit assessment is always necessary before the treatment. In our opinion, further studies are required to assess the risk-benefit ratio of preoperative embolization with new liquid embolic agents in asymptomatic vascular head and neck tumours.

## Data Availability

The datasets used and/or analysed during the current study are available from the corresponding author on reasonable request.

## Abbreviations

EVOH: Ethylene-vinyl alcohol copolymers
CT: Computed Tomography
MRI: Magnetic Resonance Imaging
CN: Cranial nerve
DMSO: Dimethyl sulfoxide
V-VST: Volume-viscosity swallow test
ECA: External carotid artery
